# Food insecurity status and mortality among adults in Ontario, Canada

**DOI:** 10.1371/journal.pone.0202642

**Published:** 2018-08-23

**Authors:** Craig Gundersen, Valerie Tarasuk, Joyce Cheng, Claire de Oliveira, Paul Kurdyak

**Affiliations:** 1 Department of Agricultural and Consumer Economics, University of Illinois, Urbana, Illinois, United States of America; 2 Department of Nutritional Sciences, University of Toronto, Toronto, Ontario, Canada; 3 Centre for Addiction and Mental Health, Toronto, Ontario, Canada; 4 Institute for Clinical Evaluative Science, Toronto, Ontario, Canada; 5 Institute of Health Policy, Management and Evaluation, University of Toronto, Toronto, Ontario, Canada; 6 Department of Psychiatry, University of Toronto, Toronto, Ontario, Canada; State University of Rio de Janeiro, BRAZIL

## Abstract

**Background:**

Food insecurity is associated with a wide array of negative health outcomes and higher health care costs but there has been no population-based study of the association of food insecurity and mortality in high-income countries.

**Methods:**

We use cross-sectional population surveys linked to encoded health administrative data. The sample is 90,368 adults, living in Ontario and respondents in the Canadian Community Health Survey (CCHS). The outcome of interest is all-cause mortality at any time after the interview and within four years of the interview. The primary variable of interest is food insecurity status, with individuals classed as “food secure”, “marginally food insecure”, “moderately food insecure”, or “severely food insecure”. We use logistic regression models to determine the association of mortality with food insecurity status, adjusting for other social determinants of health.

**Results:**

Using a full set of covariates, in comparison to food secure individuals, the odds of death at any point after the interview are 1.28 (CI = 1.08, 1.52) for marginally food insecure individuals, 1.49 (CI = 1.29, 1.73) for moderately food insecure individuals, and 2.60 (CI = 2.17, 3.12) for severely food insecure individuals. When mortality within four years of the interview is considered, the odds are, respectively, 1.19 (CI = 0.95, 1.50), 1.65 (CI = 1.37, 1.98), and 2.31 (CI = 1.81, 2.93).

**Interpretation:**

These findings demonstrate that food insecurity is associated with higher mortality rates and these higher rates are especially large for the most severe food insecurity category. Efforts to reduce food insecurity should be incorporated into broader public health initiatives to reduce mortality.

## Introduction

Food insecurity, the uncertainty or inability to acquire sufficient food because of financial constraints [1], is a growing population health concern in many high-income countries for two reasons. First, the magnitude of the problem is staggering. In 2012, 12.6% of Canadian households were affected by some degree of food insecurity; this is the highest rate observed since national monitoring began in 2007 [1]. Second, a vast literature has demonstrated the numerous negative health consequences associated with food insecurity [2]. Among adults, these include increased risk of diabetes [3,4], hypertension [4], dyslipidemia [5], cardiovascular disease [6], depression [7,8], poor sleep [9], and iron deficiency [10]. In addition, food insecurity is associated with poorer disease management and indications of greater disease severity across a broad spectrum of communicable and non-communicable diseases [11–20]. As might be expected, food insecurity is also associated with higher health care costs in Canada [20,21].

The health consequences associated with food insecurity, many of which are chronic in nature, are also associated with higher mortality rates. As a consequence, given the myriad of chronic health conditions associated with food insecurity and the greater likelihood of poor disease management in this context, one would anticipate that food insecurity is also associated with increased risk of mortality. To date this association has only been documented among specific subgroups of HIV-positive adults [13,22].

The objective of this study is to ascertain the association between food insecurity and all-cause mortality for a population-based sample of adults. Addressing this objective hasn’t been possible before due to the lack of information on mortality that can be matched with food insecurity information. To overcome this limitation and address the objective of this study, we use a unique administrative data set, which contains information on all users of medical care in Ontario, Canada, including records of death, and match those individuals who were also interviewed in the Statistics Canada’s Canadian Community Health Survey (CCHS).

## Methods

### Data sources

This study made use of data from CCHS conducted in 2005, 2007–08, and 2009–10 [23–26]. The CCHS is a repeated cross-sectional survey which is representative of 98% of the Canadian population aged 12 and over. (Individuals living on First Nations reserves, in institutions, and in the Canadian Armed forces are not part of the sampling frame.) For each household one member, 12 years of age or older, is selected for the survey based on sampling probabilities reflecting age and household composition. The CCHS data for the province of Ontario were linked to uniquely encoded identifiers from the administrative health care data housed at the Institute for Clinical Evaluative Sciences (ICES) in Toronto Ontario.

### Study population

Our initial linked sample comprised 91,752 adults who were, 18 years of age and older living in Ontario with a valid Ontario health insurance number in the 12 months prior to the CCHS interview. The exclusion of individuals with missing data resulted in a final analytic sample of 90,368 individuals.

Out of these individuals, 69,096 had information on household income. To minimize the loss of sample due to missing data on income, neighborhood-level income quintiles, derived by linking 2006 census data to the respondents’ residential postal code data, were used for subjects with missing household income [27]. We estimate models using (a) the sample which includes missing household income observations combined with the neighborhood-level income quintiles (N = 90,368) and (b) the sample which only includes those reporting household income and using household income in those models (N = 69,096).

### Measures

#### Primary exposure: Household food security status

The CCHS is used to delineate household food security status and socio-demographic characteristics. Consistent with its standard, validated approach [1], household food insecurity over the past 12 months was assessed using an 18-question module regarding food hardships. Based on these responses, we assign households to marginal, moderate and severe food insecurity categories based on Health Canada’s coding method [28]. (See Appendix A in S1 File for the 18 items and coding algorithm applied to determine household food insecurity status.)

#### Primary outcome: Mortality

The CCHS survey was linked with the Ontario Registered Persons Database, which contains information on the vital status of all Ontario residents. Our interest in this paper is in whether or not someone has died since appearing in the CCHS. We further categorize the outcome into (a) dying since interview and (b) dying within four years of the interview.

#### Other covariates

Insofar as other variables may influence mortality rates, we also include age, gender (female = 1), education level (less than secondary school graduation, secondary school graduation, some postsecondary schooling, post-secondary school completion (reference category)), homeownership (renters = 1), neighborhood-level income quintile, number of children in the household, and number of adults in the household in the models. This set of covariates was also selected to adjust for other well-established social determinants of health that are also known to be associated with household food insecurity status. Given that several years of the CCHS are being used, the probability of observing someone who has died is greater with earlier CCHS participation so we control for year of survey in our models.

### Statistical analysis

Sample characteristics are described with means and proportions. We used linear trend tests to examine the association between each covariate and food security status. (The comparison is for each category reflecting a food insecure household (marginally food insecure, moderately food insecure, severely food insecure) versus food secure.) Analysis of variance was used for continuous variables and the Cochran–Armitage test for categorical variables.

Logistic regression models, expressed in odds ratios, were used to determine the association between food insecurity status (our primary exposure measure), other covariates, and mortality. For both mortality within four years and mortality at any point after the interview, we estimate two sets of models for the probability of mortality: with the three levels of food insecurity without any covariates; and with the levels of the other covariates listed above. All analyses were conducted with the use of SAS statistical software, version 9.2. The significance levels were set at *p* < 0.05.

## Results

Concentrating on the larger sample where we use all observations, including those with missing income, the mean age of participants in the study sample was 51.3 years (standard deviation ± 18.7), 54.9% were female, the majority of the sample had completed post-secondary education (56.2%), 77.3% were homeowners, and the mean number of children was 0.4 (standard deviation ± 0.9) and adults was 2.0 (standard deviation ± 0.9) (Table 1, top panel). With respect to food insecurity status, 3.4% lived in marginally, 4.4% in moderately, and 3.2% in severely food insecure households. When stratified by household food insecurity status, with the exception of number of children when household income is used, the participants differed significantly in age, education level, home ownership, household composition, and household income (Table 1). With respect to mortality, in our sample with income quintiles, 7,810 (8.6%) of the sample had died after the survey was taken and 3,757 (4.2%) had died within four years of the survey.

**Table 1:** Characteristics and mortality status by household food insecurity status.

	All	Food secure	Marginally food insecure	Moderately food insecure	Severely food insecure	
	All Observations (N = 90,368)^a^					
Food secure	89.8	100.0				
Marginally food insecure (%)	3.4		100.0			
Moderately food insecure (%)	4.4			100.0		
Severely food insecure (%)	3.2				100.0	
Age	51.3±18.7	51.3±18.8	43.4±17.6	44.2±16.5	43.3±14.2	< .0001
Female (%)	54.9	54.2	59.0	62.1	63.0	< .0001
Less than secondary school graduation (%)	17.7	17.0	21.1	25.6	26.9	< .0001
Secondary school graduation (%)	18.6	18.4	20.9	20.1	18.3	0.018
Some postsecondary school (%)	7.5	7.2	10.2	9.6	10.6	< .0001
Post-secondary school completion (%)	56.2	57.4	47.8	44.7	44.2	< .0001
Renter (%)	22.7	18.9	42	56.9	71.3	< .0001
Number of children	0.4±0.9	0.4±0.8	0.7±1.1	0.6±1.0	0.5±0.9	0.0207
Number of adults	2.0±0.9	2.0±0.8	1.9±0.9	1.8±0.9	1.5±0.7	< .0001
First neighborhood income quintile	20.0	18.3	28.9	35.0	42.9	< .0001
Second neighborhood income quintile	20.3	20.1	22.9	23.5	21.9	< .0001
Third neighborhood income quintile	20.3	20.5	20.3	18.1	16.0	< .0001
Fourth neighborhood income quintile	19.9	20.6	15.4	13.8	10.8	< .0001
Fifth neighborhood income quintile	19.5	20.5	12.5	9.7	8.3	< .0001
Respondent has died (%)	8.6	8.8	6.6	6.9	8.1	< .0001
Respondent has died within 4 years of interview (%)	4.2	4.2	3	3.7	3.8	0.0106
	Observations with Household Income (N = 69,096)					
Food secure	89.9	100.0				
Marginally food insecure (%)	3.3		100.0			
Moderately food insecure (%)	4.6			100.0		
Severely food insecure (%)	2.7				100.0	
Age	50.3±17.8	51.1±17.9	43.5±16.8	44.6±16.0	43.4±13.7	< .0001
Female (%)	52.7	51.8	57.3	61.3	62.0	< .0001
Less than secondary school graduation (%)	16.2	15.4	20.1	24.1	26.4	< .0001
Secondary school graduation (%)	17.7	17.5	19.0	19.9	17.3	0.019
Some postsecondary school (%)	7.2	6.9	10.1	9.7	11.0	< .0001
Post-secondary school completion (%)	58.9	60.2	50.8	46.3	45.3	< .0001
Renter (%)	23.7	19.7	43.74	57.7	72.8	< .0001
Number of children	0.4±0.9	0.4±0.8	0.7±1.1	0.6±1.0	0.5±0.9	0.1664
Number of adults	1.9±0.8	1.9±0.8	1.8±0.8	1.7±0.8	1.5±0.07	< .0001
Household income	69506 ±55213	73655 ±56116	44827 ±34373	33278 ±26182	23742 ±19829	< .0001
Respondent has died (%)	8.2	8.3	6.6	6.8	8.5	0.0227
Respondent has died within 4 years of interview (%)	3.9	3.9	3	3.7	3.7	0.2357

In the bottom panel of Table 1, the sample is composed of only households which reported incomes. This results in a 23.5% decline in the sample, from 90,368 observations to 69,096 observations. The average values of the variables in the bottom panel are similar to those in the top panel. In particular, the mortality rates are similar; 8.2% had died after the survey was taken and 3.9%had died within four years of the survey.

As seen in Table 2, the probability of mortality, both within four years of interview and at any point, is lower for food insecure individuals when no other covariates are included. After controlling for age, gender, and other factors, those who are marginally food insecure are 28% more likely to die at any point after the interview than those who are food secure and the results for moderately food insecure and severely food insecure are 49% and 160%. When mortality within four years of the interview is considered, the results are, respectively, 19%, 65%, and 131%. (With the exception of marginally food insecure, each of these is statistically significant.) When the sample is truncated to only include those reporting income and then using income rather than the quintile of neighborhood income (Table 3), the results are similar to Table 2.

**Table 2:** Results of regression model predicting mortality, with neighborhood income measures (N = 90,368).

	At any Point After Interview		Within Four Years of Interview	
	Unadjusted Odds Ratio	Adjusted Odds Ratio	Unadjusted Odds Ratio	Adjusted Odds Ratio
Marginally food insecure	0.725	1.282	0.692	1.194
	(0.627, 0.839)	(1.081, 1.521)	(0.559, 0.855)	(0.95, 1.501)
Moderately food insecure	0.761	1.494	0.864	1.647
	(0.672, 0.863)	(1.292, 1.728)	(0.73, 1.022)	(1.371, 1.979)
Severely food insecure	0.912	2.598	0.901	2.306
	(0.782, 1.065)	(2.173, 3.106)	(0.723, 1.122)	(1.815, 2.929)
Age		1.109		1.097
		(1.107, 1.112)		(1.093, 1.1)
Female		0.579		0.535
		(0.548, 0.612)		(0.497, 0.575)
Less than secondary school graduation		1.344		1.423
		(1.262, 1.431)		(1.311, 1.545)
Secondary school graduation		1.181		1.194
		(1.094, 1.275)		(1.078, 1.322)
Some postsecondary school		1.147		1.258
		(1.017, 1.29 3)		(1.077, 1.469)
Renter		1.512		1.457
		(1.415, 1.617)		(1.338, 1.587)
Number of children		0.918		0.918
		(0.847, 0.996)		(0.822, 1.025)
Number of adults		0.932		0.984
		(0.893, 0.972)		(0.931, 1.04)
Second neighborhood income quintile		1.017		0.958
		(0.938, 1.103)		(0.863, 1.063)
Third neighborhood income quintile		0 .94		0.908
		(0.864, 1.022)		(0.814, 1.013)
Fourth neighborhood income quintile		0.951		0.942
		(0.873, 1.037)		(0.842, 1.053)
Fifth neighborhood income quintile		0.896		0.838
		(0.821, 0.978)		(0.747, 0.941)
Year		0.761		0.983
		(0.748, 0.774)		(0.96, 1.004)

**Table 3:** Results of regression model predicting mortality, with household income (N = 69,096).

	At any Point After Interview		Within Four Years of Interview	
	Unadjusted Odds Ratio	Adjusted Odds Ratio	Unadjusted Odds Ratio	Adjusted Odds Ratio
Marginally food insecure	0.786	1.26	0.769	1.205
	(0.666, 0.929)	(1.038, 1.53)	(0.604, 0.978)	(0.929, 1.561)
Moderately food insecure	0.806	1.317	0.934	1.51
	(0.7, 0.929)	(1.119, 1.551)	(0.772, 1.129)	(1.227, 1.858)
Severely food insecure	1.025	2.422	0.957	2.029
	(0.868, 1.211)	(1.995, 2.941)	(0.75, 1.221)	(1.554, 2.651)
Age		1.104		1.091
		(1.101, 1.107)		(1.087, 1.095)
Female		0.585		0.541
		(0.548, 0.624)		(0.496, 0.589)
Less than secondary school graduation		1.294		1.407
		(1.201, 1.395)		(1.275, 1.552)
Secondary school graduation		1.096		1.112
		(1.002, 1.199)		(0.985, 1.256)
Some postsecondary school		1.1		1.183
		(0.959, 1.263)		(0.986, 1.42)
Renter		1.415		1.384
		(1.312, 1.526)		(1.255, 1.526)
Number of children		0.941		0.921
		(0.859, 1.03)		(0.812, 1.045)
Number of adults		0.957		1.029
		(0.906, 1.01)		(0.958, 1.104)
Income/10000		0.951		0.951
		(0.940, 0.962)		(0.936, 0.966)
Year		0.766		0.993
		(0.751, 0.782)		(0.968, 1.019)

## Discussion

Our study showed that household food insecurity status has a strong association with mortality for adults in Ontario, independent of other well-established determinants of mortality. Our multivariate results demonstrate that along with the previously demonstrated wide array of negative health outcomes [2], and the higher health care costs associated with food insecurity [20,21], those who are food insecure are more likely to die than those who are food secure. The fact that mortality risk follows a food insecurity severity gradient lends further credibility to the overall food insecurity/mortality relationship.

Despite the mounting evidence of the negative outcomes associated with food insecurity, Canada has yet to make the reduction of household food insecurity a priority for policy intervention. While there are ad hoc community-based food charities and other food programs, these lack the capacity to effectively alter household food insecurity [29–32]. In Canada, as elsewhere, problems of food insecurity are tightly linked to household incomes and other measures of financial resources [1]. Policy reforms that have improved the adequacy and security of incomes of lower income, working-aged adults and their families have been shown to yield marked reductions in food insecurity [33–35]. Consistent with these findings, the government of Ontario recently implemented a three-year pilot study to assess the impact of a guaranteed minimum income for working aged adults on a variety of indicators of health and well-being, including food security [36]. Our findings highlight the importance of continued support for policy interventions to reduce food insecurity and, potentially, mortality.

One other path to reducing food insecurity is found in the United States which has had a large-scale food assistance program–the Supplemental Nutrition Assistance Program (SNAP)–for over 50 years [37]. Multiple studies have demonstrated its success in alleviating food insofar as, after controlling for non-random selection into the program, participants are substantially less likely to be food insecure. (Recent work includes, e.g., [38,39].) With respect to Canada, we estimate that adding a program like SNAP to Canada’s existing array of social programs could result in as much as a 16% decline in food insecurity [40]. Whether the introduction of a publicly-funded food assistance program would have any advantage over measures to strengthen Canada’s existing fabric of income support programs is questionable, but this finding further highlights the potential to reduce household food insecurity, and therefore potentially mortality, through interventions that improve household resources.

This is the first paper that has examined the relationship between food insecurity and mortality in a population-representative sample, with a standard food security measure. There are four limitations to this paper that can be addressed in future research. First, as the severity of food insecurity increases so too does its association with mortality. More research is needed to elucidate the mechanisms through which this occurs. Second, it isn’t clear as to whether certain causes of death are disproportionately related to food insecurity. We were limited to an examination of all-cause mortality because we lacked data on specific causes of mortality. Third, we only have information on the mortality of Ontario residents, and the relationship between food insecurity status and mortality might differ in the other provinces and territories in Canada. Linking CCHS data to health administrative data nationally would be useful to more fully examine this issue and others. Fourth, we have not made causal claims regarding the association between food insecurity and mortality and our final recommendation is to consider causal issues. This is part of a broader call to address causal issues in the food insecurity, health nexus [2].

Our finding that food insecurity has a robust association with mortality among adults in Ontario highlights the seriousness of long-observed associations between food insecurity and various negative health outcomes and the importance of policy and programmatic interventions to reduce both the prevalence and severity of household food insecurity.

## Supporting information

S1 File Appendix A: CCHS Household Food Security Survey Module. (DOCX)
